# Treatment of Tuberculosis with Mercury

**Published:** 1909-05-29

**Authors:** 


### TREATMENT OF TUBERCULOSIS WITH MERCURY.

Papers on the above subject by Surgeon Wright, of the U.S. Navy, though attracting much attention in America and describing some remarkable results, have not yet been brought under general notice in this country in any detail. By far the most striking argument is the following statement, referring to tuberculous cases at the naval hospital at New Fort Lyon, Colorado:—

Surgeon Wright's Statistics.

"During the first quarter, ending March 31, 1908, our death-rate was 11.29 per cent.; during the following quarter, ending June 30, the death-rate was 4.76 per cent. During the quarter ending September 30 we have had but one death, with a daily average of 105 patients, giving a death-rate of 0.95 per cent., or a reduction over the previous quarter of 80 per cent., and over the quarter ending March 31 of 91 per cent. . . . These results appear all the more remarkable when we consider the fact that over 60 per cent. of the patients on admission are far advanced, in many of whom grave secondary involvements complicate the serious pulmonary lesions."

The author was led to give mercury by observing rapid improvement under this drug of patients suffering from both syphilis and tubercle. But the great majority of those above referred to are vouched for by the senior medical officer of the hospital to be cases of pure tuberculosis. At first oral administration was employed, but gastric derangement caused intra-muscular injection to be substituted. Besides pulmonary cases, 15 laryngeal—10 of them ulcerative—are said to have been cured, and genito-urinary lesions to have been greatly benefited. Surgeon Wright recalls the similarity microscopically of gumma and tubercle, and advances the rather interesting speculation that Lustgarten's bacillus, which its discoverer found to disappear from the tissues of syphilitics under mercury and which resembles the tubercle bacillus closely, is really the tubercle bacillus itself.

Method of Administration.

Real interest, however, centres in the directions as to technique and dosage; for the treatment is simple and seems worth trying, and no injurious effects have yet been reported after experience extending over 1,100 injections. First of all, the usual general treatment must not be neglected. The preparation used is hydrargyri succinimidum, dissolved in distilled water, which, like the needle and syringe, is boiled for twenty minutes. The solution then made is gr. $\frac{1}{5}$ to 10 minims. The patient lies prone on a table, and the skin of the buttock is prepared with scrubbing-brush, soap, alcohol, ether, and perchloride solution. The surgeon's hands are likewise sterilised.

The hypodermic needle is driven in with a quick downward plunge. If blood issues it must be reinserted, as a vein has been entered; if not, the syringe is connected and the drug injected. The dosage is an injection every other day, beginning with gr. $\frac{1}{10}$, and increasing gradually to the therapeutic limit, perhaps gr. $\frac{3}{5}$. The dose is then halved, and injections continued until thirty have been given. Next follows two weeks' rest from all medication, and then injections are resumed. Indications to stop or restrict treatment are sore gums, or a slight rise in temperature, or loss of weight or appetite. The treatment must also be modified to suit individual cases.

